# Mosquito Gut Bacterium May Curb Malaria

**DOI:** 10.1289/ehp.119-a337

**Published:** 2011-08-01

**Authors:** Harvey Black

**Affiliations:** Harvey Black of Madison, WI, has written for *EHP* since 1994. His work has also appeared in *Environmental Science & Technology*, *ChemMatters*, and the *Milwaukee Journal Sentinel*.

Before *Plasmodium falciparum*, the most important human malaria parasite, can infect people, it must first infect its mosquito host. But that’s not always so easy to do, according to a team of researchers from the Johns Hopkins University Bloomberg School of Public Health and the Malaria Institute at Macha in Zambia. The team reports that a bacterium naturally found in mosquitoes produces a short-lived molecule that can kill the *P. falciparum* parasite in its early stages of life.^1^ “These bacteria could be used for the development of a biocontrol strategy [to combat malaria],” says senior author George Dimopoulos, an associate professor in the Johns Hopkins University Department of Molecular Microbiology and Immunology.

Dimopoulos and his colleagues isolated bacteria from the midgut of wild *Anopheles arabiensis* mosquitoes collected in Zambia, then fed the bacteria to specimens of the main malaria-transmitting mosquitoes in Africa (*An. gambiae*) and Asia (*An. stephensi*). They observed that certain Gram-negative bacteria inhibited the early development of *P. falciparum* in these mosquitoes. One newly discovered member of the *Enterobacter* genus, *Esp_Z*, almost completely curtailed the development of *P. falciparum* ookinetes, oocysts, and sporozoites *in vitro*. Further study indicated the bacterium’s effectiveness was the result of its production of reactive oxygen species, which were previously known to kill *P. falciparum*. The study was partially funded by the National Institute of Allergy and Infectious Diseases.

John Beier, a professor of epidemiology and public health at the University of Miami who was not involved in the research, calls the finding a “significant discovery.” Beier says being able to deliver such a bacterium to wild mosquitoes may decrease the rate of transmission of malaria in humans. Existing strategies to combat malaria—including insecticide-treated bed nets, indoor spraying of insecticides, and efforts to eliminate mosquito breeding sites such as stagnant water—are not sufficient to eliminate the disease, according to Beier. In the most recent figures available, the World Health Organization reports there were 247 million cases of malaria in 2008 and nearly 1 million deaths, mostly among children in Africa.^2^

**Figure:**
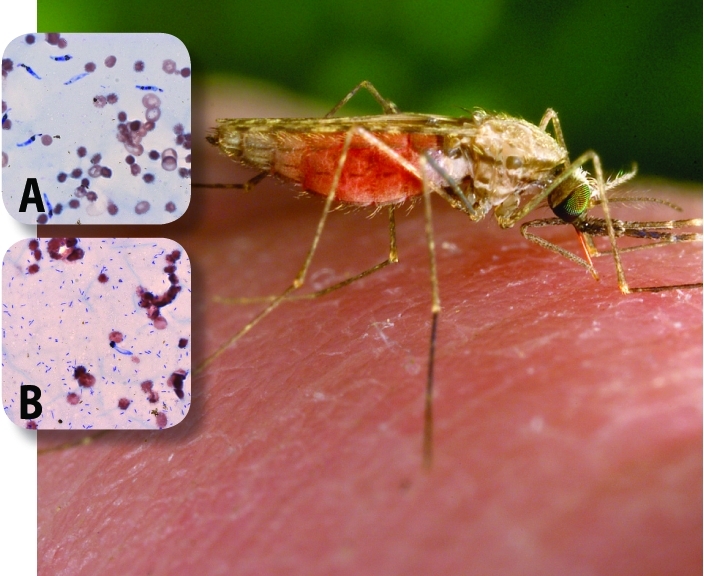
Panel A shows P. falciparum parasites (elongated bodies stained in blue) that have developed in the absence of Esp_Z, a newly discovered member of the Enterobacter genus. Panel B shows that parasite numbers decrease drastically in the presence of Esp_Z (small blue-stained rods). Jim Gathany/CDC; insets: Yuemei Dong

Dimopoulos says one potential method of using these bacteria to fight malaria would be to capitalize on mosquitoes’ hunger for sugar. Whereas female mosquitoes need blood only for the development of eggs, males and females must feed on sugar every day; plant nectar is their food of choice. “We could mix the bacteria into an artificial nectar and spray it on vegetation,” Dimopoulos says, “or we could develop so-called feeding stations to attract the mosquitoes, where they would feed on nectar and pick up the bacteria as well.”

Similar techniques are being tested in other countries. In Mali, researchers reported that spraying local plants with a bait of fermented fruit juice mixed with boric acid reduced the population of *An. gambiae* by 90% at one study site.^3^ A study in Israel demonstrated that when sugar water spiked with the insecticide spinosad was sprayed on the fruit trees where mosquitoes normally fed, the mosquito population was virtually wiped out.^4^^,^^5^

Beier notes more research is needed to understand mosquitoes’ sugar-feeding behavior in nature—something he himself is studying. He also says there should be an effort to find more of the sort of inhibitory bacteria that Dimopoulos has identified. “I think there is a need to demonstrate how such bacteria would work in multiple mosquito species. Wherever there is malaria transmission, you have a number of different competent vector species,” he says.

Dimopoulos and his colleagues reported finding *Esp_Z* in only 25% of the mosquitoes sampled.^1^ He says this may reflect the vagaries of sampling, or perhaps not all mosquitoes are exposed to the bacterium or the bacterium may have trouble colonizing the mosquito gut. He and his colleagues are working to help the bacterium better establish itself in the mosquito gut. “Through many, many rounds of selection, one can adapt this bacterium to become better at colonizing the mosquito,” Dimopoulos says.

## References

[1] Cirimotich CM (2011). Natural microbe-mediated refractoriness to *Plasmodium* infection in *Anopheles gambiae*.. Science.

[2] WHO. World Health Organization Media Centre Malaria Fact Sheet. Geneva, Switzerland:World Health Organization (April 2010). Available: http://tinyurl.com/yolhob [accessed 27 Jun 2011]

[3] Muller GC (2010). Successful field trial of attractive toxic sugar bait (ATSB) plant-spraying methods against malaria vectors in the *Anopheles gambiae* complex in Mali, West Africa.. Malaria J.

[4] Muller G, Schlein Y. (2006). Sugar questing mosquitoes in arid areas gather on scarce blossoms that can be used for control.. Int J Parasitol.

[5] The authors observed that other insects in the study area apparently ingested spinosad, but they did not comment on how these insects were affected.

